# Challenging One Model With Many Stimuli: Simulating Responses in the Inferior Colliculus

**DOI:** 10.3813/aaa.919249

**Published:** 2018

**Authors:** Langchen Fan, Kenneth S. Henry, Laurel H. Carney

**Affiliations:** 1) Department of Biomedical Engineering, University of Rochester, New York, USA; 2) Department of Otolaryngology, University of Rochester, New York, USA; 3) Department of Neuroscience, University of Rochester, New York, USA.

## Abstract

Existing models to explain human psychophysics or neural responses are typically designed for a specific stimulus type and often fail for other stimuli. The ultimate goal for a neural model is to simulate responses to many stimuli, which may provide better insights into neural mechanisms. We tested the ability of modified same-frequency inhibition-excitation models for inferior colliculus neurons to simulate individual neuron responses to both amplitude-modulated sounds and tone-in-noise stimuli. Modifications to the model were guided by receptive fields computed with 2nd-order Wiener kernel analysis. This approach successfully simulated many individual neurons’ responses to different types of stimuli. Other neurons suggest limitations and future directions for modeling efforts.

## 1. Introduction

The inferior colliculus (IC) is a critical center in the auditory system – all ascending pathways converge in the IC, en route to the thalamus and cortex [1]. Neurons in the IC are rate-tuned to sinusoidally amplitude-modulated (AM) sounds, as described by modulation transfer functions (MTFs, discharge rates vs. AM frequency). IC neurons with band-enhanced (BE) MTFs have increased rates when their neural inputs contain fluctuations near the best modulation frequency (BMF); those with band-suppressed (BS) MTFs have decreased rates for a range of fluctuation frequencies.

Adding a tone to narrowband Gaussian noise flattens the stimulus envelope [2], as reflected in peripheral responses [3]. Therefore, in response to tone-in-noise (TIN) stimuli, band-enhanced IC neurons are expected to have decreasing response rates with addition of a tone, and band-suppressed IC neurons are expected to have increasing rates. These predicted changes in rate in response to TIN stimuli based on MTF types are consistent with neural responses of many BE and BS neurons, but not all [4]. Computational models may provide insights into these complex neural responses.

Many existing models have been developed to explain human psychophysical results and responses of different auditory neurons. These models are usually based on a single stimulus type; however, in general the same neurons respond to many different stimulus types. Attempting to simulate neural responses to different stimulus types with one model will better explain how single neurons function and provide better population simulations in the future. This study was an initial step in this direction. The same-frequency inhibition-excitation (SFIE) model has previously been shown to simulate IC responses to AM sounds at different modulation frequencies for both BE and BS MTF shapes [5, 6]. We generalized the SFIE model by varying the frequency tuning of neural inputs, input delays, and the number of inputs to match the response characteristics of single neurons. These modifications were guided by the neuron’s receptive field obtained from the Fourier transform of the 2nd-order Wiener kernel. Each neuron-specific model was then tested for its ability to simulate the neuron’s responses to AM stimuli, TIN stimuli, and white noise. For this initial study, the inputs to the model were provided by an AN model for cat [7], as a detailed model for the rabbit periphery is not available.

## 2. Methods

### 2.1. Physiological methods

Extracellular recordings were made with tetrodes in the IC of awake Dutch-belted rabbits and sorted offline to isolate single units. The current study focused on twenty neurons for which the pattern of excitation and inhibition in the receptive field (RF) could be readily used to specify the model structure and parameters (see below).

Detailed physiological methods are described in Carney et al. [8]. Briefly, neural responses to pure tones from 250Hz to 20kHz were recorded to determine characteristic frequency (CF). Neural responses to sinusoidal AM noise and tones, TIN stimuli, and long-duration white noises were recorded. AM tones had carrier frequencies at CF, modulation frequencies from 2 to 350Hz (for low CFs) or 1024Hz (for high CF neurons) and were presented at 70dB SPL. AM wideband noise (0.1–10kHz) had modulation frequencies from 2 to 350Hz and was presented at a spectrum level of 30dB SPL. All AM stimuli had 1-sec durations. TIN stimuli had a tone near CF and 1/3-octave bands of noise centered near CF, with 0.3-sec durations. Overall noise level for TIN stimuli varied from 35 to 75dB SPL in 10-dB steps; tones were presented at signal-to-noise ratios (SNRs) ranging from −12dB to 8dB. White noises (0.1–20kHz) were 2-sec in duration, presented at 65dB SPL.

**Figure 1.**
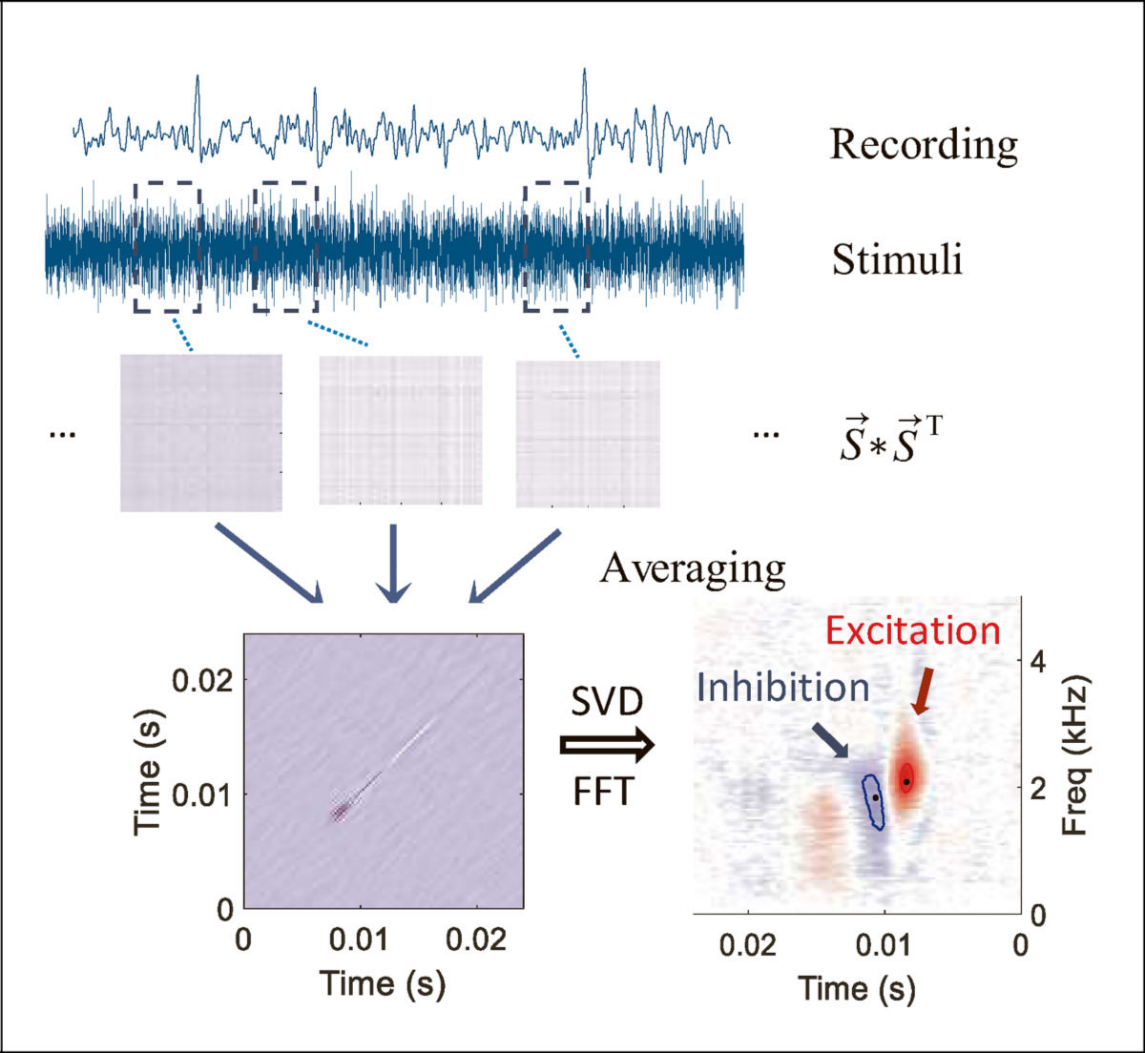
(Colour online) Calculation of the receptive field (RF) using the 2nd-order Wiener kernel. The kernel was calculated by averaging the product of instantaneous spike rates with the outer product of pre-spike stimulus epochs. A 1-D Fourier transform yielded the RF. Singular-value decomposition (SVD) was used to identify excitation and inhibition in the RF [9].

Average discharge rates were computed for responses to AM sounds and TIN stimuli. Second-order Wiener kernels were calculated by multiplying the instantaneous spike rates and the outer product of time-reversed pre-spike stimulus segments [9] (Figure 1). The 1-D Fourier transform of the kernel provides an estimate of the neuron’s RF. Excitation and inhibition in the RF can be identified with singular value decomposition [9]. Smoothing and peak-finding algorithms were applied to determine the center frequency and latency of the excitation and inhibition. Second-order Wiener-kernel derived RFs are an alternative to spectrotemporal RFs [10, 11].

### 2.2. Modeling methods

**Figure 2.**
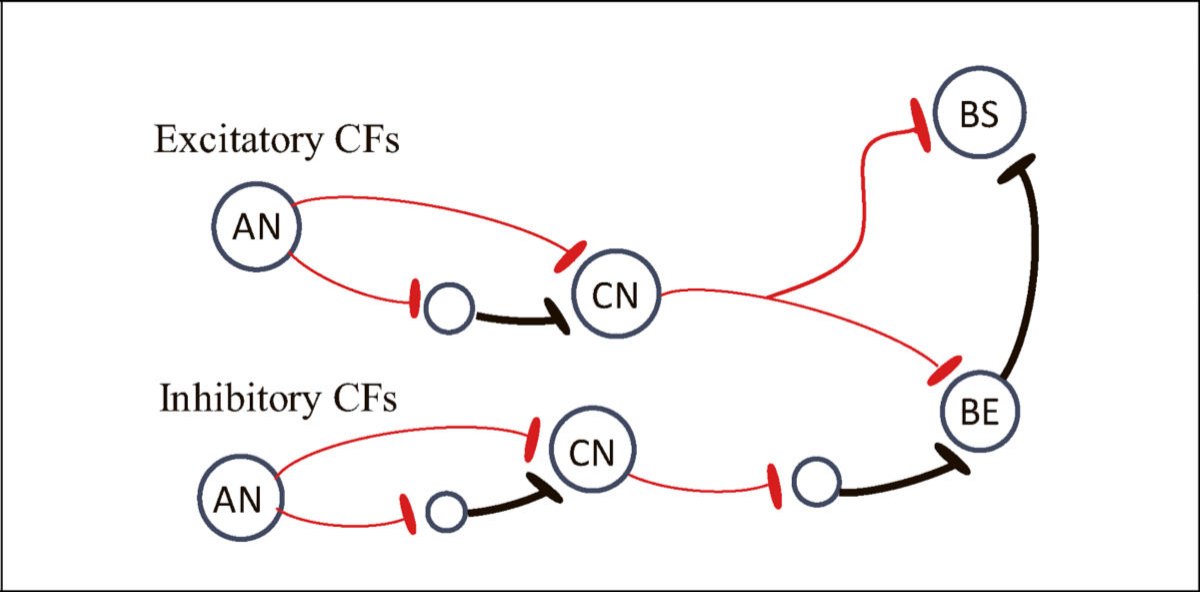
(Colour online) Illustration of generalized SFIE model. The auditory nerve (AN) model provides excitatory (red, thin line) and inhibitory (black, thick line, via an interneuron) inputs to the cochlear nucleus (CN) model. The CFs and number of excitatory and inhibitory inputs to the inferior colliculus (IC) are not limited, but for simplicity, this diagram includes only one of each. The band-enhanced (BE) IC neuron receives excitatory and inhibitory inputs from CN; the band-suppressed (BS) IC neuron receives excitatory input(s) from the CN and is inhibited by a BE neuron.

The original SFIE model is comprised of a model of an auditory-nerve (AN) fiber [7], brainstem (cochlear nucleus, CN) and IC neurons [5, 6]. The CN model always receives excitatory and delayed inhibitory inputs from a single AN model and, therefore, has the same CF as the AN model. The IC neurons in the SFIE model have one excitatory and one delayed inhibitory input. Here, we generalized the SFIE model by removing restrictions on the CFs and numbers of excitatory and inhibitory inputs (Figure 2). Relative timing between the first strong excitation frequency (f_exc1_) and other excitation or inhibition frequencies (f_exc2_, f_inh1_ and in some cases f_inh2_) were identified from the RF.

Latencies based on the RF were used for all modified IE models. Four variations of the model were considered: a) one excitatory and one inhibitory input with the same CF; b) one excitatory and one inhibitory input with different CFs, based on the RF; c) a second inhibitory input, with CF matched to the excitatory input, was added to b); and d) up to two excitatory and two inhibitory inputs with CFs selected based on the RF. Responses were also simulated with the original SFIE model as a reference. The strength of the inhibitory (str_inh_) and second excitatory (str_exc2_, when present) inputs to the IC were adjusted relative to the strongest excitatory input (str_exc_) based on the number of excitatory and inhibitory inputs: for modifications a) and b), str_inh_ = 1.3; for c), str_inh_ = 0.8 (both inhibitory inputs); for d), str_exc_ = 1, str_inh_ = 1.3 when two excitatory and inhibitory inputs were used; str_exc1_ = 1, str_exc2_ = 0.8, str_inh_ = 2.2 for two excitatory and inhibitory inputs; str_inh_ = 0.8 for one excitatory and two inhibitory inputs. Note that once the parameters for a given model were selected, the model was fixed to simulate responses to all stimulus types.

White noises used for simulations had the same statistics and parameters as in the neural recordings. AM sounds and TIN stimuli for simulations were identical to those used for recordings. Sound levels of all stimuli used as model inputs were reduced by 10dB, because the models were slightly more sensitive than the neurons. Model performance was evaluated using Pearson correlations between model and neural MTFs and TIN responses. For TIN, correlation coefficients were calculated for each overall noise level. Internal noise was contributed by the random variations in the AN model responses; no additional internal noise was added.

## 3. Results

**Figure 3.**
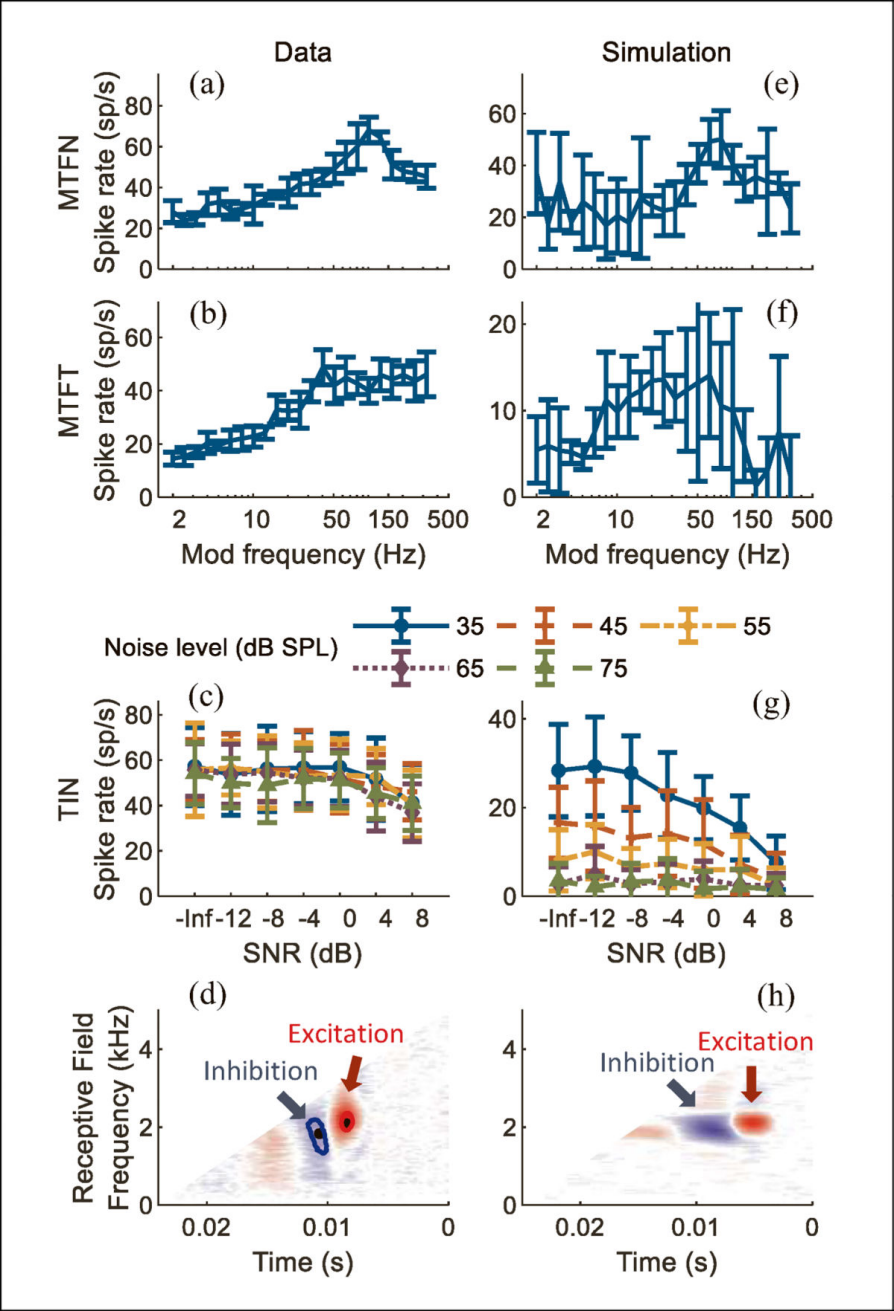
(Colour online) Example of a band-enhanced IC neuron. MTFs (rate vs. modulation frequency) for a) noise and b) tone carriers; errorbars show standard deviation. c) Neural responses to TIN stimuli (rate vs. SNR) for several noise levels. d) Receptive field (time reversed) calculated with the 2nd-order Wiener-kernel analysis Ű the center of excitation and inhibition used in modified models are marked by black dots; e) to h): simulations with modified IE model for the same stimuli as in a)–d).

Figure 3a–d shows an IC neuron with a band- enhanced MTF. The neuron’s TIN responses decreased with increasing SNRs, consistent with the prediction based on MTF type. Model results (modification b) are shown in Figure 3e–h. Excitatory and inhibitory frequencies used in the model were 2083Hz and 1833Hz, respectively; the inhibitory input was delayed by 2.3ms. Simulations for both MTFs and TIN stimuli followed the trends in neural responses. The correlation between MTF data and simulation was significant for noise carrier (*r* = 0.68, *p* < 0.001) but not for tone carrier (*r* = 0.19, *p* = 0.19). For TIN stimuli, correlation coefficients were calculated for each noise level. Correlation coefficients for five overall noise levels (from low to high) were: 0.82, 0.96, 0.90, 0.51, 0.65; p values were 0.01, <0.001, 0.04, 0.12, 0.058, respectively. Correlations were significant for all datasets except the TIN responses at the two highest noise levels. The model receptive field (RF) had excitatory and inhibitory regions that were similar to the neural RF (Figure 3d and 3h).

**Figure 4.**
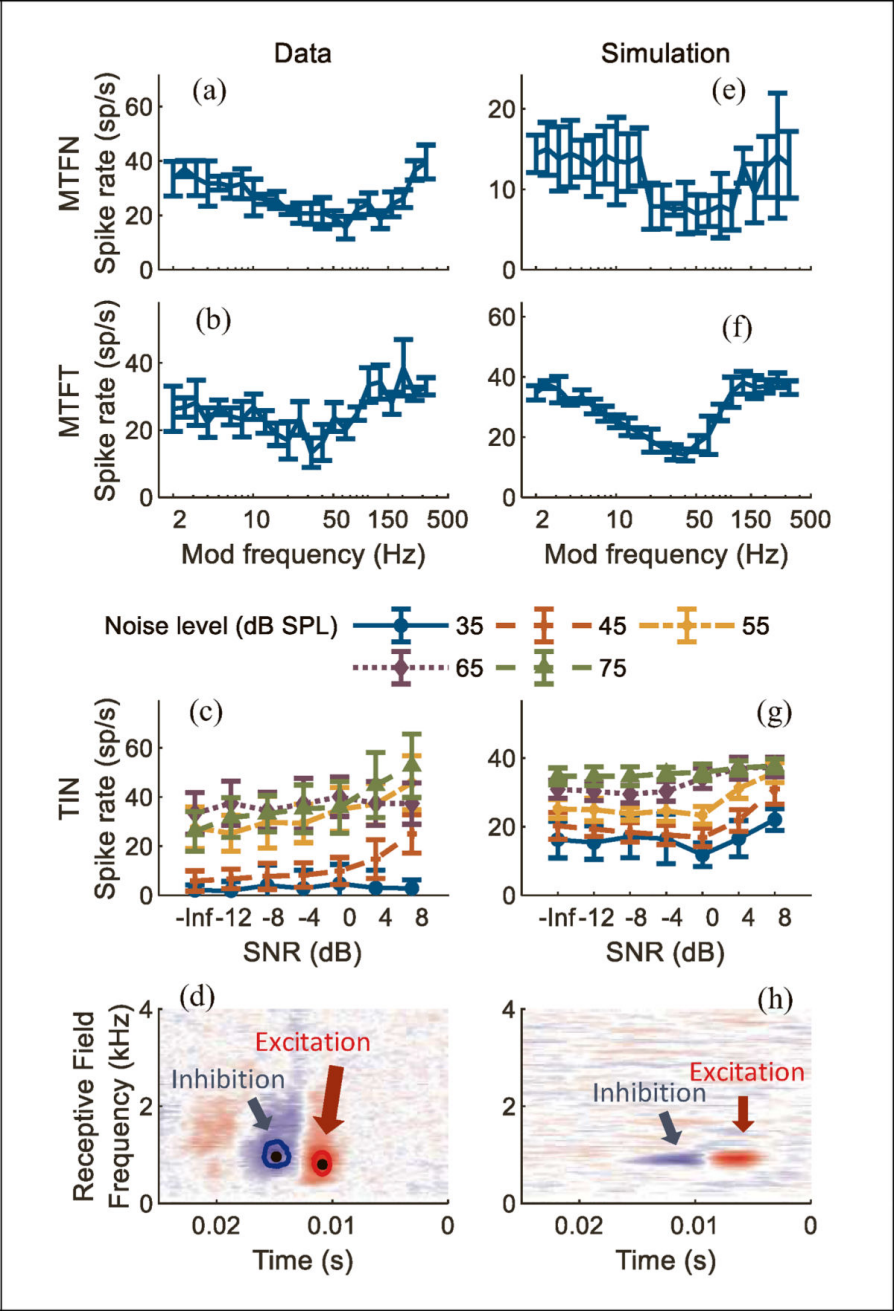
(Colour online) Example of a band-suppressed IC neuron. Format same as Figure 3.

Figure 4 shows an IC neuron with band-suppressed MTF for both noise and tone carriers (a and b). The neuron’s TIN response rates increased with increasing SNRs, consistent with the prediction for this MTF type. Model results (modification c, one excitation and two inhibitions) are shown in Figure 4e–h. The excitation CF was 800 Hz; one inhibition at 800 Hz and one at 960 Hz were used. Both inhibitory delays were 4 ms. Simulations of this neuron’s MTFs successfully replicated both the shape and the lowest point in the band-suppressed MTF. The correlations between MTF data and simulations were significant for both noise carrier (*r* = 0.77, *p* < 0.001) and tone carrier (*r* = 0.82, *p* < 0.001). For TIN stimuli, correlation coefficients for five overall noise levels (from low to high) were: −0.37, 0.91, 0.84, 0.46, 0.96; *p* values were 0.80, 0.002, 0.008, 0.15, <0.001, respectively. The model RF had similar excitatory and inhibitory frequencies as the neural RF, but missed some details. For example, the frequency range of excitation and inhibition were much wider in the neural RF than in the model RF. The simulated 2nd-order Wiener kernel had inhibition at frequencies higher than the excitation, possibly due to high-frequency suppression in the model AN responses. In this example neuron, the simulated kernel also had excitation at frequencies higher than the inhibition. This pattern was possibly related to the model structure: band-suppressed MTFs were the result of inhibition from a band-enhanced neuron (Figure 2); therefore, inhibition of the BE neuron facilitated responses of the BS neuron.

To determine whether modification significantly improved model performance, a paired t-test was performed between the results of the SFIE model and results of each of the modified models for each stimulus type. For the TIN stimuli, the average correlation coefficient across five noise levels was used in the test. None of the modified models had significantly higher predictive value than the original SFIE model for any stimulus type. Thus, the performance of the general model structure was relatively robust, and it was not strongly influenced by fine-tuning the model parameters based on the RF of an individual neuron. Because internal noise was included in the AN model, correlations between model and neural responses varied slightly across simulations and models. The percentage of simulations that were significantly correlated to multiple responses was calculated based on average results of two rounds of simulations with the five models described above. Model responses were significantly correlated to a single neuron’s response to at least one type of MTF (with tone or noise carrier, or both) and at least three noise levels for TIN in 42.7% of cases. Model responses were significantly correlated to both types of MTF and at least three TIN levels in 17.4% of cases.

## 4. Discussion

An SFIE-type model with fixed parameters can simulate a single neuron’s responses to different stimulus types, for IC neurons with both band-enhanced and band-suppressed MTFs. The shape and best-modulation frequency of the MTF could be replicated for many neurons in the current study. In response to tone-in-noise stimuli, trends in the modeled responses with increasing tone level at different overall noise levels agreed with the neural responses for many neurons. For responses to white noise, the general pattern of excitation and inhibition was simulated, but discrepancies between the simulated and neural responses require further study.

Although the SFIE model was originally developed to understand modulation tuning in IC neurons, there were some neurons for which the MTFs were not successfully simulated. This discrepancy might be due to differences between the frequency and latency picked from the receptive field and the set of values used to describe the standard BE MTFs in the original SFIE model. Large changes in these parameters may not be compatible with the simple structure of the SFIE model; for example, large changes in the latency of inhibition can result in discontinuities in the SFIE model’s impulse response.

In response to TIN stimuli, simulated rates decreased more strongly with increasing overall noise level than was observed in IC BE neurons. This decrease in rate in response to TIN stimuli is influenced by saturation of the inner hair cell in the AN model [3], thus differences between simulated and neural responses may be due to differences between AN model properties and the rabbit AN responses (see below).

Failure to predict some neural responses could be due to limitations in interpreting patterns of the receptive field. We used the centers of the excitation and inhibition RF areas to specify the frequency and latency of model inputs. However, in many cases, the excitation and inhibition spanned a wider frequency range in the RF, which may reflect wider tuning in the rabbit periphery in comparison to the cat tuning in the AN model. Whether the bandwidth of peripheral filters plays a role in these modeling results could be tested in the future using an AN model adapted to the rabbit. Also, 2nd-order Wiener kernel analysis only shows the “net” excitation or inhibition at one frequency and time. Therefore, the frequency and timing parameters estimated from the RF for use in the IE models may not be accurate estimates of the underlying excitatory and inhibitory response components.

Patterns of excitation and inhibition in the RF can be complicated. In most neural RFs, excitation has a shorter latency than inhibition. However, in a small number of neurons that were not included in this study, the latency of the inhibition was shorter than that of excitation. Simply reversing the order of the excitation and inhibition in the models used here did not yield successful simulations of these neurons. Other neurons (not included) had RFs with several excitatory and inhibitory bands from which model parameters were not easily specified. The complex patterns in the RFs provide a strong challenge for simplified neural models of the type used here.

Our results also show that the SFIE model without modifications performed approximately as well as the modified models. Thus, although the SFIE model performance was relatively robust, it was not sensitive to changes in the input frequencies and latencies based on the RF that were hypothesized to improve simulations. Alternative models that combine excitation and inhibition should be explored in the future, including coincidence detectors that receive excitatory and inhibitory inputs [12].
